# Neural encoding of biomechanically (im)possible human movements in occipitotemporal cortex

**DOI:** 10.1371/journal.pcbi.1013694

**Published:** 2025-12-08

**Authors:** Giuseppe Marrazzo, Federico De Martino, Albert Mukovskiy, Martin A. Giese, Beatrice de Gelder

**Affiliations:** 1 Department of Cognitive Neuroscience, Faculty of Psychology and Neuroscience, Maastricht University, Maastricht, The Netherlands; 2 Center for Magnetic Resonance Research, Department of Radiology, University of Minnesota, Minneapolis, Minnesota, United States of America; 3 Hertie Institute for Clinical Brain Research and Center for Integrative Neuroscience, University Clinic Tübingen, Tübingen, Germany; Universite Paris Cité, FRANCE

## Abstract

Understanding how the human brain processes body movements is essential for clarifying the mechanisms underlying social cognition and interaction. This study investigates the encoding of biomechanically possible and impossible body movements in occipitotemporal cortex using ultra-high field 7T fMRI. By predicting the response of single voxels to impossible/possible movements using a computational modelling approach, our findings demonstrate that a combination of low-level, postural, biomechanical, and categorical features significantly predicts neural responses in the ventral visual cortex, particularly within the extrastriate body area (EBA), underscoring the brain’s sensitivity to biomechanical plausibility.

## Author summary

How does the human brain know whether a body movement is physically possible or not? To answer this, we used ultra-high-field 7T fMRI to record brain activity while participants watched short videos of human-like avatars performing either natural, biomechanically plausible actions or subtly “impossible” variants. We then applied computational models to predict each voxel’s response. Across the ventral occipitotemporal cortex, and especially within the extrastriate body area (EBA), a mixture of low-level motion cues, postural keypoints, graded biomechanical distances, and simple possible/impossible tags together explained over 10% of the BOLD signal variance. Our findings highlight that body-selective visual regions are sensitive to biomechanical plausibility.

## Introduction

Human bodies convey essential information about others’ actions, intentions, and emotions and provide critical cues in social communication [1–4]. Previous research using functional magnetic resonance imaging (fMRI) to investigate the neural basis of body perception has primarily focused on localizing high-level visual category-specific representations. Specific regions in the occipitotemporal and fusiform cortex are selectively responsive to images of bodies, the extrastriate body area (EBA) and the fusiform body area (FBA) [5,6]. Similar findings of distinct body sensitive patches were found in monkeys in the ventral bank of the superior temporal sulcus (STS), namely the middle STS body patch (MSB) and the anterior STS body patch (ASB), with a putative homology between MSB and EBA, and ASB and FBA [7]. When dynamic images or functional aspects of body perception like action and emotional expression are also considered, body sensitivity was reported in other areas [8]. This has raised interest in investigating the neural mechanisms underlying body sensitivity, notably in the specific computational mechanisms operating across these different body sensitive areas.

Some studies argued that EBA is more involved in processing body parts and local features and FBA devoted to holistic processing [9,10]. There is also some evidence that EBA and FBA might process a combination of local and global body features [11–14], depending on semantic attributes such as emotion and action [15–17], and that EBA is sensitive to task demands [18]. Additionally, recent findings further suggest that activity in the Default Mode Network (DMN) is sensitive to the contrast between biological and non-biological motion based on the naturalness of kinematic patterns. Specifically, the DMN’s stronger response to human-like motion, particularly when it matches expected kinematics, suggests that it may modulate or support EBA and FBA processing by enhancing sensitivity to motion patterns that carry social and biological relevance [19].

However, despite these insights, there is no clear understanding of a functional division of labour between different body-sensitive areas. A better understanding of the computational processes within these body-selective areas should clarify their specific contributions to body perception.

Over the past decade, (linearized) encoding [20,21] has been used to compare different computational hypotheses of brain function. In these approaches, brain activity (e.g., blood oxygen level-dependent (BOLD) signals in a voxel or brain region during fMRI) is predicted based on stimulus features derived from computational models. The accuracy of these predictions can then be compared to adjudicate between competing models, or to determine the relative contribution (the variance explained) of each model [22–28]. Encoding models predict neural responses based on specific stimulus features and have been successfully applied to visual processing in early visual cortex [20,21] as well as higher visual cortex [29,14,25,30]. An earlier study used encoding models to human body-selective regions [14] and shed light on the relevance of joint positions and their spatial configuration for the responses in the EBA to still images. Like most prior research in the field, the use of still images, only addressed postural aspects rather than movement, thus limiting our understanding of how the brain processes more complex, dynamic information.

Here, we probed EBA’s dependency on joints configuration by using biomechanical manipulations of natural movements based on 3D motion capture (mocap) data. Creating videos that disrupt the natural spatial configuration of joints allowed us to investigate how EBA processes biomechanical plausibility. This approach is particularly important with moving bodies, as dynamic stimuli capture the temporal and kinematic properties essential for understanding how the brain encodes real-world, biologically relevant movements. We specifically tested the hypothesis that EBA is sensitive to biomechanical characteristics of body movements, building on some earlier indications in the literature. For instance, participants exhibit automatic imitation effects even for impossible movements, indicating the brain’s predisposition to process action dynamics despite biomechanical violations [31]. Recognition of human bodies is significantly affected by inversion, reflecting specialized perceptual mechanisms for recognizing human shape in upright configurations [32]. More recent studies have shown that prior knowledge of biomechanical constraints biases visual memory, with participants misremembering extreme postures as less extreme, adjusting their perceptions toward more biomechanically plausible positions [33]. Developmental evidence also points to an early sensitivity to biomechanical constraints on human movement. 12-month-old infants as well as adults spend more time looking at the elbows during impossible arm movements compared to possible ones [34], and newborns can differentiate between biomechanically possible and impossible hand movements [35]. Investigating the neural correlates of humanly impossible movements has further revealed that impossible finger movements elicit distinct neural responses compared to possible ones in EBA [36]. The influence of biomechanics on processing of visual information related to the body may be fundamental to how body representations are formed in the brain.

To investigate the computations underlying the neural responses to body movements in the occipitotemporal cortex, we utilized ultra-high-field 7 Tesla fMRI and linearized encoding models, assessing macroscopic and mesoscopic (layer-specific) responses related to biomechanical sensitivity. We aimed to identify how different cortical layers within the EBA encode biomechanical information and distinguish between possible and impossible movements. We employed four distinct encoding models to probe these computations: the 3D Keypoints (kp3d) model, which represents three-dimensional coordinates of body joints and captures precise postural information; the Similarity Distances (SimDist) model, which quantifies biomechanical differences between possible (natural) and morphed (impossible) movements based on motion capture data [37]; the categorical differences model, which provides a higher-level distinction by categorizing movements as biomechanically possible or impossible; and a motion energy model, which implements a dense bank of spatiotemporal Gabor filters to capture low-level dynamic cues across the visual field [38].

Together, these four models span a hierarchy of hypotheses: from pure low-level motion filtering (motion energy), through body pose encoding (kp3d), to graded biomechanical deviation (SimDist), up to a binary plausibility distinction (categorical differences). By jointly fitting all feature spaces, we can assess how much each contributes uniquely to the representation of dynamic body movements in occipitotemporal cortex as well as investigating its sensitivity to body plausibility.

## Materials and methods

### Ethics Statement

All experimental procedure conformed to the Declaration of Helsinki and the study was approved by the Ethics Committee of the faculty of Psychology and Neuroscience of Maastricht University. Before the experiment, all participants provided written informed consent, indicating their voluntary agreement to participate in the study.

### Participants

Twelve right-handed volunteers (five males; mean age 27.8 ± 3.8 years) were recruited from the Maastricht University student and staff cohorts. All participants reported normal or corrected-to-normal vision and no history of neurological or psychiatric disorders. One participant was excluded from the main analysis for excessive head motion across multiple runs. All subjects were naïve to the task and the stimuli and received monetary compensation for their participation. Scanning sessions took place at the neuroimaging facility Scannexus at Maastricht University (NL).

### Main experiment stimuli

**Fig 1:**
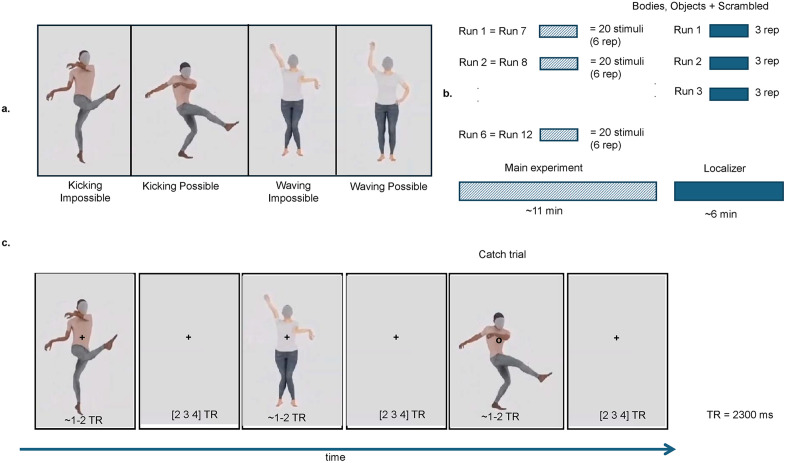
Stimuli and experimental procedure. **(a)** The videos were generated by animating mocap data from the MoVi dataset [37]. Sixty possible videos were created from 17 actors performing 4 actions: kicking, jumping, pointing, waving. Additionally, we modified the joint angles of the elbows and knees to create 60 biomechanically impossible videos. In panel (a) we show frame of possible videos and their equivalent impossible. **(b)** For each run 1/6 of the stimuli (20) where presented in a pseudo-randomized order following a fast event-related design. Each stimulus was repeated three times per run. Each run was repeated two times across sessions resulting in a total of 120 stimuli repeated six times. To identify body sensitive region, the localizer stimuli included videos of humans performing natural body movement, objects, and their scrambled version. We presented stimuli following a block-design with each block repeated three times per run. **(c)** During the main experiment participants fixated on the cross and were presented with the stimuli depicting possible and impossible body movement for 1-2 TRs (depending on the length of each video) followed by a blank screen which appeared for 2, 3 or 4 TRs. When the fixation cross turned to a circle, they had to press a button whether with the right index finger. TR = 2300ms.

The stimulus set consisted of 120 videos of two avatars (1 male and 1 female). The videos were generated by animating mocap data from the MoVi dataset [37], which includes recordings from 60 female and 30 male actors performing 21 daily actions and sports movements. For this experiment, we animated six specific actions (kicking, pointing, waving, jumping, jumping jacks, and walking sideways) performed by 17 actors (9 males). The movements of these 17 actors were then used to animate the two avatars, ensuring that the presented stimuli maintained diversity in motion while being standardized in appearance. This process resulted in 96 videos depicting natural body movements. Additionally, we modified the joint angles of the limbs to create 96 biomechanically impossible videos. To refine the set for the fMRI experiment, we conducted a behavioral validation, to select stimuli showing the greatest difference between possible and impossible movements. This ultimately reduced the set to 120 videos (60 possible videos created from 17 actors performing 4 actions: kicking, jumping, pointing, waving). More details are provided in the behavioral validation section below. Each video was edited to have a length between 60 and 90 frames, corresponding to 2–3 seconds at 30 frames per second. Additionally, the avatars in each video were aligned to be centered relative to the fixation cross, ensuring a consistent starting position across all videos. During the experiment, the stimuli spanned a mean width and height of 1.84º x 4.32º of visual angle (Fig 1a).

### Localizer stimuli

Stimuli for the localizer experiment consisted of videos depicting two object categories: bodies, objects. Additionally, also a scrambled version of each stimulus was included. (Fig 1b). The size of the stimuli was 3.5 * 7.5 degrees for human bodies and objects. For more details about the localizer stimuli we refer to [39]. None of the stimuli from the localizer were used in the main experiment.

### Behavioural validation

The stimuli created from the mocap data comprised 96 videos of natural body movements (possible) and their corresponding modified versions, for a total of 192 stimuli. These modified versions (impossible) were created by altering the joint angles of the limbs to produce biomechanically impossible movements. We violated the anatomical constraints of the elbows and knees, by mirroring those joints orientations for each time point of a trajectory. Accordingly, we modified the shoulders and wrist joint angles, as well as ankles and hips, in order to preserve the end-effectors (hands and feet) orientations to be as close as possible to the original (possible) ones for every time point.

Out of the total 192 videos, we selected 120 (60 possible and their impossible version) for the fMRI experiment through a process of behavioral validation. This selection was based on identifying the stimuli that best demonstrated the intended differences between possible and impossible movements, ensuring the most effective set for the experiment. We asked 136 participants (25 males, mean age = 21.45 ± 2 years) to rate the stimuli using a questionnaire consisting of two Likert-scale questions and one categorical question. Participants were presented with half (96) of the total stimuli (192) once. For each participant, the stimuli were pseudo-randomized (96 stimuli randomly selected for each participant, but evenly distributed so that each stimulus was rated by approximately the same number of participants: mean number of responses = 68 ± 2.24). After each presentation, participants were asked to answer a total of three questions about the plausibility/realism of the body movement, action content and salience of specific body parts (see S1 Text).

### MRI acquisition and experimental procedure

Participants viewed the stimuli while lying supine in the scanner. Stimuli were presented on a screen positioned behind participant’s head at the end of the scanner bore (distance screen/eye = 99 cm) which the participants could see via a mirror attached to the head coil. The screen had a resolution of 1920x1200 pixels, and its angular size was 16º (horizontal) x 10º (vertical). The experiment was coded in Matlab (v2021b The MathWorks Inc., Natick, MA, USA) using the Psychophysics Toolbox extensions [40,41,42].

Each participant underwent two MRI sessions, we collected a total of twelve functional runs (six runs per session) and one set of anatomical images. Images were acquired in a 7T MR scanner (Siemens Magnetom) using a 32-channel (NOVA) head coil. Anatomical (T1-weighted) images were collected using MP2RAGE MP2RAGE: 0.7 mm isotropic, repetition time (TR) = 5000 ms, echo time (TE) = 2.47 ms, matrix size = 320 x 320, number of slices = 240. The functional dataset (T2*-weighted) covered the occipitotemporal cortex and was acquired using a Multi-Band accelerated 2D-EPI BOLD sequence, multiband acceleration factor = 2, voxel size = 0.8 mm isotropic, TR = 2300 ms, TE = 27 ms, number of slices = 58 without gaps; matrix size = 224 x 224; number of volumes = 300, GRAPPA factor = 3. In addition to functional images, phase images were simultaneously acquired along with five noise volumes appended at the end of each run.

During the main experiment, stimuli were presented on the screen for 2–3 seconds (depending on the length of each video) with an inter stimulus interval that was pseudo-randomised to be 2, 3 or 4 TRs. Participants were asked to fixate at all times on a white cross at the centre of the screen (Fig 1c).

To control for attention, participants were asked to detect a shape change at the fixation cross (cross to circle) and respond via button press with the index finger of the right hand. Within each run, 20 stimuli (10 possible and 10 impossible) were presented and repeated 3 times. Three target trials were added for a total of 63 trials per run. The two sessions were identical therefore each of the 120 videos was repeated 6 times (3 repetitions x 2 sessions) across the 12 runs. Additionally, three blank trials were added in each run lengthening the baseline period.

Across sessions, we collected 2–3 runs of localizer depending on available scanning time. Each localizer run contained 10 videos per category presented following a block design. Each block lasted 25 seconds (10 videos x 1 sec + 1.5 sec intertrial interval) and was followed by a jittered fixation period of 11 seconds on average. Each category block was repeated 3 times per run. During the localizer participants performed the same task as in the main experiment.

Preprocessing for the functional images was performed using BrainVoyager software (v22.2, Brain Innovation B.V., Maastricht, the Netherlands), Matlab (v2021b) and ANTs [43]. To lower thermal noise, we performed NOise reduction with DIstribution Corrected (NORDIC) using both magnitude and phase images [44]. EPI Distortion was corrected using the Correction based on Opposite Phase Encoding (COPE) plugin in BrainVoyager, where the amount of distortion is estimated based on volumes acquired with opposite phase-encoding (PE) with respect to the PE direction of the main experiment volumes [45], after which subsequent corrections is applied to the functional volumes. Other preprocessing steps included scan slice time correction using cubic spline, 3D motion correction using trilinear/sinc interpolation and high-pass filtering (GLM Fourier) cut off 3 cycles per run. During the 3D motion correction process, all runs were aligned to the first volume of the first run using the scanner’s intersession auto-align function, ensuring consistent spatial alignment across sessions. Anatomical images were resampled at 0.4mm isotropic resolution using sinc interpolation. To ensure a correct functional-anatomical and functional-functional alignment, the first volume of the first run was coregistered to the anatomical data in native space using boundary based registration [46]. Functional images were exported in nifti format for further processing in ANTs. To reduce non-linear intersession distortions, functional images were corrected using the antsRegistration command in ANTs using as target image the first volume of the first run and as moving image the first volume of all the other runs. Volume Time Courses (VTCs) were created for each run in the normalized space (sinc interpolation). Prior to the encoding analysis (and following an initial general linear model [GLM] analysis aimed at identifying regions of interest based on the response to the localizer blocks), we performed an additional denoising step of the functional time series by regressing out the stimulus onset (convolved with a canonical hemodynamic response function [HRF]) and the motion parameters. This step was crucial for minimizing the influence of external confounds, such as the timing of stimulus presentation and participant head motion, on the neural data. By removing these factors, we ensured that the model’s training focused exclusively on learning patterns directly associated with the features of the encoding models. However, this approach, while effective in isolating feature-driven neural responses, can lead to smaller accuracies as it also removes some of the variance explained by the stimulation paradigm itself. Despite this trade-off, this method provides a cleaner and more specific evaluation of the encoding models’ ability to capture the relevant neural patterns.

Segmentation of white matter (WM) and gray matter (GM) boundaries as well as cortical layers estimation was performed using a custom pipeline. First, the UNI image and T1 image obtained from MP2RAGE were exported to nifti. We performed gaussian noise reduction using the DenoiseImage command in ANTs [47], and bias field correction in SPM12 as described on layer fMRI blog (https://layerfmri.com/2017/12/21/bias-field-correction/). After preprocessing of anatomical images, cortical reconstruction and volumetric segmentation was performed using Basic SAMSEG (cross-sectional processing) command of the Freesurfer image analysis suite (http://surfer.nmr.mgh.harvard.edu/), using the UNI images as T1w contrast and the T1 map of the MP2RAGE (which has flipped intensities between white and gray matter, resembling a T2w image) as T2w contrast. Lastly, cortical thickness and layers extraction were performed using surf_laynii.sh script (https://github.com/srikash/surf_laynii/blob/main/surf_laynii) which enables layering in LAYNII [48] using the Freesurfer segmentations output. Three layers were then calculated in LAYNII using the equi-volume approach. All analyses were performed in the individual subject space, but for visualization purposes we projected single-subject statistical or encoding maps onto a group cortex-based aligned surface and then averaged the results across subjects [49]. By matching the folding geometry rather than relying solely on volume landmarks, this approach reduces anatomical variability and enhances statistical sensitivity [50] (see section on Statistical Analysis for more details).

### Voxel selection for encoding analysis

The functional time series of the localizer runs collected in each participant were analysed using a fixed-effect GLM with 5 predictors (4 conditions in the localizer: Body Objects and their scrambled version and 1 modelling the catch trials). Motion parameters were included in the design matrix as nuisance regressors. The estimated regressor coefficients representing the response to the localizer blocks were used for voxel selection. A voxel was selected for the encoding analysis if significantly active (q(FDR)<0.05) in response to the Body and Objects categories. Note that this selection is unbiased to the response to the stimuli presented in the experimental section of each run.

### Functional ROI definition

Using the functional localizer we also defined body selective regions at the single subject level. Specifically, the EBA was defined using the contrast [Body + Body Scrambled]> [Objects + Objects Scrambled] [51] with a statistical threshold of q(FDR) < 0.05. All subsequent ROI-level analyses were conducted by identifying the intersection between the voxels assigned to the EBA and those selected for the encoding analysis.

### Encoding models

In order to understand what determines the response to body images we tested several hypotheses, represented by different computational models, using fMRI encoding [52,20,21,26]. We compared the performance (accuracy in predicting left out data) of four encoding models.

The first model represented body stimuli using the position of joints in three dimensions (kp3d) using 71 keypoints (main skeleton joints like hips, knees, shoulders, elbows, hands and facial features like eyeballs, neck and jaw) extracted from the MoVi dataset. This model represents the stimuli as a collection of points in space forming a human skeleton. To focus on joints that significantly influence perception while minimizing variability from less relevant keypoints, we excluded constant (or almost constant) keypoints ending up with a subset that included 56 keypoints (shoulders, elbows, wrists, hips, knees, and ankles, hands, fingers and facial features from both sides of the body).

The second model quantifies the similarity distances (SimDist) between morphed movements (impossible) and normal movements (possible) by analyzing motion capture data extracted during stimulus creation. For each video, both the modified and original motion data were loaded. Initially, all 71 joints defined in the MoVi skeleton were considered. However, to focus on joints with meaningful movement and reduce variability from less relevant joints (such as fingers and toes), joints without rotation data (i.e., joints with empty rotation indices) were excluded, reducing the original set to 56 keypoints (the same as in the previous paragraph). For each selected joint at each time frame, we converted the original Euler angles representing the joint rotation to axis-angle representation. This process yielded a set of three-dimensional vectors in Euclidian space representing the rotation of each joint over time. To measure the similarity between test movements (both modified and original) and the manifold of normal (original) movements, a Gaussian kernel-based approach was employed. This method quantifies the proximity of motion data in the high-dimensional joint angle space, allowing for a robust assessment of movement similarity (see S1 Text). Keypoints for which the computed similarity distances to the normative manifold were not finite (e.g., containing NaN or Inf values) were identified and excluded to maintain data quality, reducing the original 56 keypoints to 29. Similarity distances for all joints were then concatenated to form feature vectors representing each movement’s similarity across all considered joints. This model encoded biomechanical differences because it evaluates the kinematic properties of human joint movements by measuring their distances to a manifold of normal actions, thereby allowing for the differentiation between biomechanically plausible (possible) and implausible (impossible) movements, with the latter exhibiting higher distances due to their deviation from typical human motion patterns. Accordingly, the SimDist models tests the hypothesis that occipitotemporal regions do not exclusively tag a pose as possible versus impossible (the categorical model; see below) but rather scale their responses with the magnitude of biomechanical deviation from a normative movement manifold, allowing to ask whether a brain region codes “how impossible” a configuration is, not just that it is impossible (for the mathematical formulation see S1 Text). The third model encodes categorical differences between possible and impossible stimuli by incorporating two features that explicitly indicate the (im)possibility of each stimulus. Unlike the other models, this approach does not account for variations within each category, focusing instead on the binary classification of stimuli as either possible or impossible. This model is considered more abstract (or higher-order) compared to the kp3d and SimDist models, as it goes beyond image computable approaches (like keypoints) and instead recapitulates a conceptual distinctions. The last model is a motion energy model whose features were computed following the approach of [38] to capture low‐level spatiotemporal information from each body‐movement video. In brief, each stimulus video was first converted to its luminance channel (CIE L*A*B). We then convolved every frame with a fixed bank of spatiotemporal Gabor filters tuned to a range of orientations (0°, 45°, 90°, 135°), spatial frequencies (0.5–8 cycles/° in logarithmic steps), temporal frequencies (1–16 Hz), and motion directions (two opposite directions per orientation). Filters were implemented in quadrature pairs so that, for each channel, motion energy was computed as the sum of squares of the two phase‐offset outputs. This produced 3,703 motion‐energy channels per video, each reflecting the strength of local oriented motion at a particular scale, speed, and direction. To stabilize the dynamic range, the raw energy values were log‐transformed, and then averaged over all frames of the video.

### Banded ridge regression and model estimates

In the context of fMRI, the linearized encoding framework typically uses L2-regularized (ridge) regression to extract information from brain activity [53]. This method is effective for improving the performance of models with nearly collinear features and helps minimize overfitting. When dealing with multiple encoding models, ridge regression can either estimate parameters for a combined feature space or for each model separately. However, using a single regularization parameter for all models may not be optimal due to varying feature space requirements. To address this, banded ridge regression optimizes separate regularization parameters for each feature space, enhancing model performance by reducing spurious correlations and ignoring non-predictive features. [23,25]. In the present work we used banded ridge regression to fit the three encoding models, combined in a joint encoding model, and performed a decomposition of the variance explained by each of the models following established procedures [23,14].

Model training and testing were performed in cross-validation (3-folds: training on 8 runs [80 stimuli repeated 6 times] and testing on 4 runs [40 repeated 6 times]). For each fold, the training data were additionally split in training set and validation set (4-folds: train on 6 runs [60 stimuli repeated 6 times] and test on 2 runs [20 stimuli repeated 6 times]). Within the training set a combination of random search and gradient descent [23] was used to optimize the model fit to the data (regularization strength and model parameters). Ultimately, the best model over the 4 validation folds was selected to be tested on the independent test data (4 runs). Within each fold, the models’ representations of the training stimuli were normalized (each feature was standardized to zero mean and unit variance withing the training set). The feature matrices representing the stimuli were then combined with the information of the stimuli onset during the experimental runs. This resulted in an experimental design matrix (nrTRs x NrFeatures) in which each stimulus was described by its representation by each of the models. To account for the hemodynamic response, we delayed each feature of the experimental design matrix (5 delays spanning 11.5 seconds). The same procedure was applied to the test data, with the only difference that when standardizing the model matrices, the mean and standard deviation obtained from the training data were used.

We used banded ridge regression to determine the relationship between the features of the encoding models (stimulus representations) and the fMRI response at each voxel. The encoding was limited to voxels that significantly responded to the localizer stimuli (p(FDR)<0.05) in each individual volunteer’s data. For each cross-validation, we assessed the accuracy of the model in predicting fMRI time series by computing the correlation between the predicted fMRI response to novel stimuli (4 runs, 40 stimuli) and the actual responses. The accuracies obtained across the three folds were Z-transformed and then averaged. To obtain the contribution of each of the models to the overall accuracy we computed the partial correlation between the measured time series and the prediction obtained when considering each of the models individually [23].

### Statistical analysis

Group-inference was performed via non-parametric testing (see below) on cortex-based aligned maps (CBA) [49]. CBA begins by converting each subject’s reconstructed folded cortex into a spherical surface, carrying over sulcal and gyral curvature on the sphere. An iterative registration non-rigidly warps each individual’s curvature map against a group‐average template, thereby bringing homologous sulci and gyri into precise correspondence across participants [50,49].

We projected each subject’s native‐space encoding maps onto their own aligned surface via direct sphere-to-sphere mapping, preserving the fine‐grained topography of EBA. Statistical significance of the resulting group‐averaged CBA-aligned maps was assessed using a subject‐wise sign-flipping permutation test (2¹¹ = 2048 permutations) on the surface, with FDR correction (q < 0.05) to control for multiple comparisons. In parallel, we extracted each participant’s mean R² within their individually defined EBA ROI for the inner, middle, and superficial layers, and assessed systematic differences across depths using paired-samples t-tests. Finally, to compare the variance explained by our four models within EBA, we conducted a three-way repeated-measures ANOVA and followed up significant main effects with paired-sample t-tests.

## Results

### Consistent behavioral categorization of possible and impossible stimuli

The analysis of the questionnaire responses showed that all stimuli were accurately categorized. In the “possible” condition, each stimulus received the highest rating, confirming correct classification. Results for the “impossible” videos showed more variability while consistently scoring below 4 on the 1–7 Likert scale. Notably, 95% (57 out of 60) of these stimuli had a median rating between 1 and 2, with the remaining three videos rated between 2 and 3 (see S1 Text for more information).

### Localizer stimuli reveal activation in ventral visual cortex and EBA for voxel selection

**Fig 2:**
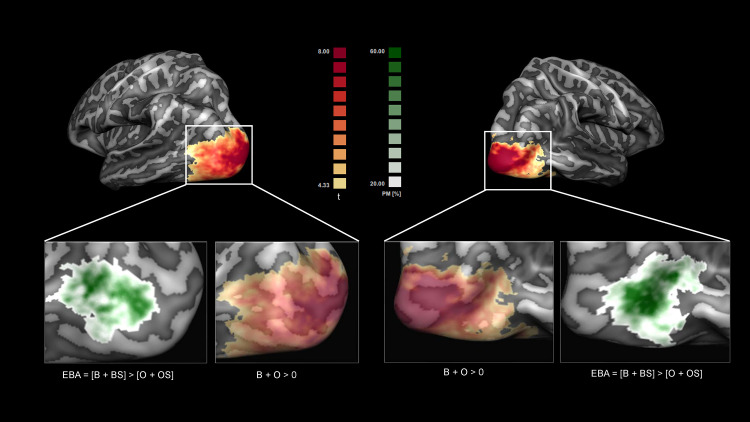
Voxels selection and EBA definition. Voxels that were significantly (q(FDR)<0.05) responding to localizer stimuli [Body + Objects]>0 were selected for the encoding analysis. Although the analysis was performed at single-subject level, for visualization purposes we show the average t-map (in red-yellow) obtained by averaging the thresholded single-subjects maps projected on a group cortex-based aligned mesh. EBA was defined within the localizer via the contrast [Body + Body Scramble]> [Objects + Objects Scramble]. Shown in white-green is a probabilistic map indicating the overlap between individually defined EBAs (q(FDR)<0.05).

In each subject, voxels that significantly responded to the localizer conditions (Body + Objects) with a false discovery rate (FDR) of less than 0.05 were selected for the encoding analysis. While selection took place at the individual level, in Fig 2 we report group-level maps obtained by averaging each subject’s thresholded (q < 0.05 FDR) single-subject maps, illustrating the approximate brain regions chosen for our subsequent encoding analyses across participants. All group maps are displayed on the group-aligned (cortex-based aligned, CBA) surface. The localizer conditions consistently activated regions in the occipitotemporal cortex, specifically in the superior, middle, and inferior occipital gyri (SOG/MOG/IOG), fusiform gyrus (FG), lingual gyrus (LG)

middle temporal gyrus (MTG), inferior temporal sulcus (ITS), lateral occipital sulcus (LOS), and superior temporal sulcus (STS). These clusters overlap with areas identified in our previous study [14]. By subtracting the responses to object stimuli from the responses to body stimuli, we defined the extrastriate body area (EBA) in each individual and computed probabilistic maps of the overlap of EBA across individuals in cortex based aligned space. The EBA spanned the MOG, MTG, and ITS (Fig 2) with the probabilistic maps showing an overlap between 20 (white in the Fig 2) and 100% (Green) of subjects.

### The joint encoding model significantly predicts responses to novel stimuli in ventral visual cortex

**Fig 3:**
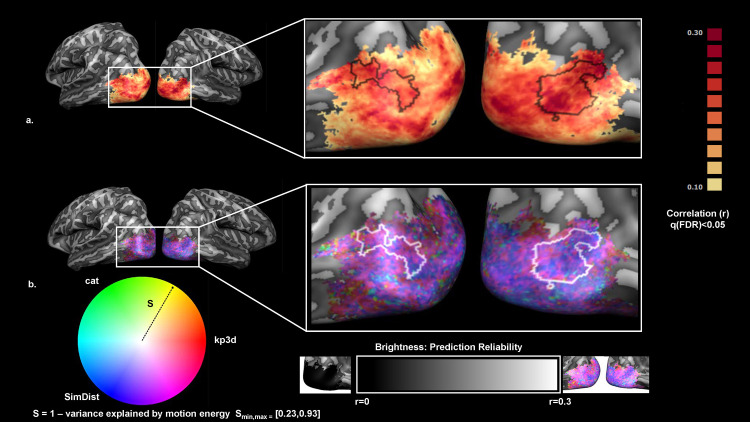
Group-level encoding results and residual variance partition (Joint model – MotEn). **(a)** Group Prediction accuracy for the joint model (kp3d, categorical, SimDist, MotEn). Statistical significance was assessed via permutation test (subject wise sign-flipping, 2^N = 2048 times with N = 11), and correction for multiple comparison was performed using FDR (q < 0.05). **(b**) Composite HSV map visualizing the residual variance partition among three feature models 3D keypoints (kp3d, red), categorical (cat, green), and similarity distances (SimDist, blue)—after factoring out motion energy. Here, hue encodes the relative proportions of the remaining variance explained by each feature model (pure red = kp3d, pure green = cat, pure blue = SimDist, and intermediate hues their mixtures), saturation (colorfulness) reflects the residual strength depicted as the radius in the hue circle S = 1 – motion energy variance (S_min_ = 23%, S_max_ = 93%) so that regions where motion energy leaves more variance appear more vivid, and value (brightness) corresponds to prediction reliability (Pearson’s r) on the same scale as in panel **(a)**. For clarity, we overlay the outline of EBA as defined in the probabilistic map depicted in Fig 2 by selecting vertices shared by at least 40% of the subjects.

The main effect of the responses in the localizer (objects + bodies) was used to select voxels for the encoding in the individual subjects’ data. In these voxels, the response elicited by body stimuli in the main experiment, independent of the localizer, was modelled using banded ridge regression. The group performance of the joint encoding model (kp3d, categorical, SimDist, MotEn) is shown in Fig 3a. Statistical significance at the group level was assessed via a permutation test, with correction for multiple comparisons using FDR (q < 0.05). The joint encoding model significantly predicted responses to novel stimuli throughout the ventral visual cortex (SOG, MOG, IOG, ITG, MTG, FG, LOS)

Spatial differences in model contributions were visualized with a single composite HSV map (Fig 3b), in which hue encodes the relative residual contributions of the three higher‐level feature models (red = kp3d; green = categorical; blue = SimDist) after factoring out motion energy, saturation (colorfulness) reflects the total residual strength (1–motion‐energy fraction), and brightness indicates overall prediction reliability (*r*). Overall, vertices are rendered in vivid magenta and purple tones reflecting that kp3d and SimDist on average jointly dominate the remaining variance. Nonetheless, ventral occipital regions show light blue/green tints (often pale) where categorical structure contributes more strongly, either alone or in combination with the SimDist model. More faded colors mark vertices where motion energy explains most of the variance, and brighter pixels highlight regions of highest model reliability. For completeness, in Fig 5 we show the amount of variance explained by the motion energy model which appear to sit between 30–40% (a) as well as its complement (b).

### EBA encodes low-level, postural, biomechanical and categorical information

**Fig 4:**
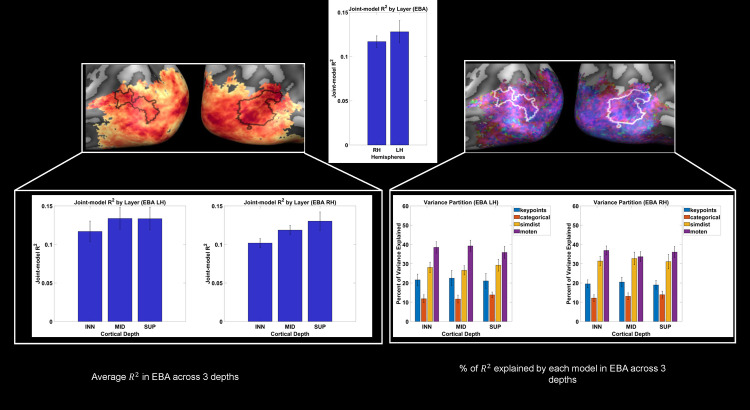
Joint model performance and variance partitioning in EBA across cortical depths. Variance partitioning in the extrastriate body area (EBA) across 11 subjects, comparing left (LH) and right hemispheres (RH) across three cortical layers (Left to right ◊ inner, middle, superficial). The top panel shows the group average R² values in the EBA, indicating overall joint model performance across hemispheres. The bottom left panels display the variance explained (R²) in the LH and RH EBA across layers. The bottom right panel illustrates the percentage of R² explained by each model across layers. To check for differences in variance explained between models, we ran a three-way repeated-measures ANOVA which showed a significant main effect of models (F(3,30)=15.59, p < .001, η²_p_ = .609).

Within the EBA, the joint encoding model accounted for approximately 10–12% of the variance of the BOLD signal (Fig 4, top panel). When considering the layer‐wise R² within EBA (Fig 4 bottom left panels), only the right‐hemisphere inner>middle contrast reached significance (t(10)=–4.01, q = 0.007, dz = 1.21), while the inner>superficial comparison narrowly missed the FDR cutoff (t(10)=–2.46, q = 0.051, dz = 0.74); no depth contrasts were significant in the left hemisphere. To test for differences in variance explained across hemisphere, layers and models (Fig 4 bottom right panels) we ran a three-way repeated-measures ANOVA which showed a significant main effect of models (F(3,30)=15.59, p < .001, η²_p_ = .609) indicating that the four feature-spaces differ substantially in the proportion of variance they explain. No other significant main effects or interactions were found. The main effect of models was further unpacked with paired-sample t-tests corrected for multiple comparison using FDR (alpha = 0.05).

In the right hemisphere, pairwise model comparisons showed that SimDist explained significantly more variance than Keypoints (t(10)=–2.65, q = 0.036, dz = 0.80) and Categorical (t(10)=–5.15, q = 0.001, dz = 1.55), and that Motion Energy outperformed both Keypoints (t(10)=–4.99, q = 0.001, dz = 1.50) and Categorical (t(10)=–7.11, q < 0.001, dz = 2.14). In the left hemisphere, Motion Energy explained more variance than Keypoints (t(10)=–2.91, q = 0.031, dz = 0.88), SimDist outperformed Categorical (t(10)=–5.00, q = 0.002, dz = 1.51), and Motion Energy again exceeded Categorical (t(10)=–9.00, q < 0.001, dz = 2.71).

These results confirm that both the biomechanical similarity-distance model and especially the motion-energy model capture substantially more variance in EBA than the simpler Keypoints and Categorical descriptors. The full tables depicting the results from the statistical analysis can be found in Tables A and B in S1 Text.

## Discussion

The present study investigated how dynamic body stimuli, specifically biomechanically possible and impossible movements, are encoded in occipitotemporal cortex. Specifically, we compared the predictive performance of encoding models based on 3D keypoints, similarity distances, categorical differences and motion energy (kp3d, SimDist, categorical, MotEn). At the group level, we observed that a combination of the four models significantly predicted fMRI BOLD responses in the ventral visual cortex after applying permutation testing and correcting for multiple comparisons. The variance partitioning across the different models of body posture in EBA across cortical layers revealed significant differences between models. In the both hemispheres, the MotEn accounted for approximately 35–40% of the joint model prediction, the SimDist 30%, kp3d 20% and categorical 10–15%, with this pattern observed consistently across cortical depths (see Fig 4).

### Low-level and high-level features in the occipitotemporal cortex

Our findings reveal that a combination of low and high-level features contribute to the dynamic perception of body movement in occipitotemporal cortex. Low-level spatiotemporal filters (MotEn) explain roughly 30–40% of the variance. Once these motion energy signals are factored out, the remaining activity is best predicted by postural (kp3d) and biomechanical (SimDist) descriptors, either alone or in combination (red, blue, and magenta–purple patches in Fig 3b). These results align with the notion that early visual areas process low-level features such as orientation, spatial frequency, and basic shape attributes [20,21,54,55,38]. As processing advances to higher visual areas, we still observe an overall predominance of kp3d/SimDist with few patches where the categorical model becomes more important either alone or in combination with the SimDist model (green, cyan patches in Fig 3b). This shift aligns with previous literature showing that higher-order areas integrate lower-level features into more abstract representations [29,56,57,58].

### Encoding of body stimuli in EBA

**Fig 5:**
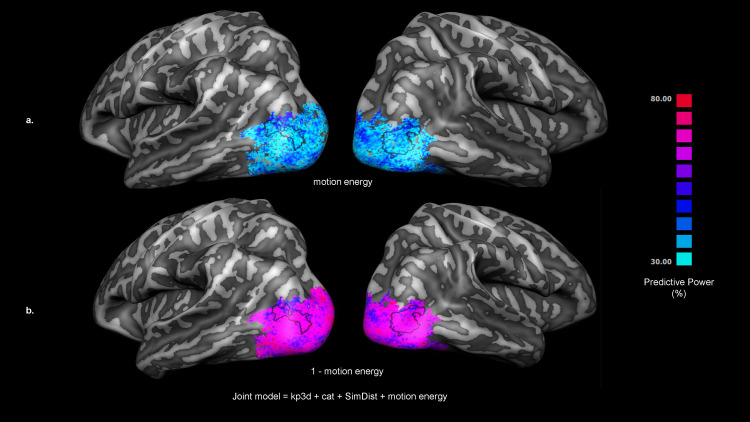
Motion-energy prediction and residual joint model performance. **(a)** Prediction power (%) of the motion-energy model alone, plotted on an inflated right hemisphere and thresholded at 30%–80% variance explained (see colorbar). **(b)** Residual prediction power, defined as the variance explained by the full joint model (kp3d + categorical + SimDist + motion energy) minus that explained by motion energy alone (i.e., 1 – motion-energy fraction). For clarity, we overlay the outline of EBA as defined in the probabilistic map depicted in Fig 2 by selecting vertices shared by at least 40% of the subjects.

Within the EBA, our joint encoding model (kp3d + SimDist + categorical + MotEn) accounted for about 10–12% of the BOLD response variance (Fig 4, top). When we partitioned that joint prediction, low-level motion‐energy filters (MotEn) explained roughly 30–40% of the variance (Fig 5a), with the remaining 60–70% of the joint‐predicted signal broken down into biomechanical similarity (SimDist ≈ 30%), 3D postural keypoints (kp3d ≈ 20%), and categorical distinctions (≈ 10–15%). In other words, MotEn, SimDist, and kp3d together capture about 85–90% of the joint model’s explanatory power, leaving the final 10–15% to semantic category information.

While low-level motion energy signals account for roughly 30–40% of the variance in EBA (Fig 5a), the remaining activity shows a clear spatial gradient in feature contributions. In the superior portion of EBA, encompassing the middle and superior occipital gyri (MOG/SOG), biomechanical similarity (SimDist) dominates the representation together with the kp3d model (purple-magenta patches in Figs 3b and 4). After controlling for low-level motion energy, high-level categorical distinctions become more prominent in the anterior ventral visual cortex, particularly across aITG and aLOS (cyan-green patches in Figs 3b and 4), suggesting an integration of postural cues with more abstract representations. This may involve linking specific body configurations to semantic information such as the type of action being performed or the emotional state conveyed by the body movement [59,60].

Our results aligns with findings that identify distinct body-selective areas within the occipitotemporal cortex [61]. Recent results by Li et al. [62] using data-driven methods identified four adjacent body-selective nodes within the occipitotemporal cortex further support this notion. Specifically, the predominance of kp3d and SimDist in superior subregions may reflect their role in detailed sensory processing, as they show stronger connectivity with regions involved in processing fine-grained visual details [62]. Our findings thus reinforce the notion that EBA is functionally heterogeneous consistent with the finding of specialized subregions dedicated to different aspects of body and action perception [62].

Furthermore, our results are consistent with previous findings showing that EBA is more functionally and structurally connected to dorsal stream regions compared to other body-related areas, such as FBA and the lateral occipital complex (LOC) [63]. This connectivity supports EBA’s role in bridging perceptual and motor functions, particularly in specifying goal-directed postural configurations for motor planning. EBA’s connectivity with parietal regions, such as the superior parietal lobule and postcentral gyrus, may enable it to access somatosensory information, which is essential for planning and executing actions based on body information. This suggestion is consistent with the earlier findings from Astafiev et al. [64] reporting that the EBA responds to goal directed movements of the observers’ body parts. Additionally, another study used real-action fMRI and multivoxel pattern analysis to show that hand-selective clusters in lateral occipitotemporal and intraparietal regions automatically distinguish typical from atypical tool grasps, even without an explicit grasping task [65]. They demonstrate that body-selective visual areas encode not only static form but also sensorimotor affordances during real interactions, underscoring the dual perceptual-motor role of these regions. Consistent with this view, another study showed that the lateral occipitotemporal cortex (LOTC) encodes abstract, object-independent representations of actions that generalize across perceptual features and task demands. This finding suggests that LOTC contributes to conceptual action understanding beyond low-level visual motion processing. [66].

### Layer-specific encoding in EBA

Layer-specific analysis in EBA revealed a subtle depth gradient in overall fit: in the right‐hemisphere joint-model performance rose from inner to middle (t(10)=–4.01, q = 0.007, dz = 1.21), while the inner to superficial comparison narrowly missed the FDR cutoff (t(10)=–2.46, q = 0.051, dz = 0.74), whereas the left hemisphere showed no significant depth dependence. Importantly, however, variance partitioning of that joint prediction remained remarkably uniform across all layers and both hemispheres: motion energy consistently explained ~35–40% of the variance, biomechanical similarity (SimDist) ~30%, 3D keypoints (kp3d) ~20%, and categorical distinctions ~10–15%, with no systematic laminar shifts (no significant main effect of layers). This depth-invariant profile may indicate that EBA’s microcircuits implement a distributed integration of low-level spatiotemporal features and higher-level postural, biomechanical, and semantic signals in parallel, rather than segregating them into distinct laminar streams [67,68,69]. Recent findings corroborate this notion as higher-level visual areas (V5/hMT+), closely adjacent to the EBA, also exhibits depth-invariant columnar tuning, indicating that microcircuits combine multiple feature representations simultaneously across all layers of the cortical ribbon [70]. Functionally, this uniform integration across depths enables downstream areas to flexibly draw on precise kinematic information for trajectory decoding or on semantic action categories for emotion recognition, in line with unified models of biological motion processing [71]. EBA’s laminar invariance in feature integration may reflect the complex requirements of dynamic body perception, which depend on the smooth fusion of motion dynamics, posture configuration, and interpretive meaning.

### Role of biomechanical plausibility

The substantial predictive power of the SimDist model from early to high-level visual cortex underscores the visual system’s sensitivity to biomechanical plausibility from the initial stages of processing. This model compares movements to a set of natural human motions. By analysing how closely a movement aligns with the typical range of human joint motions, the model allows to identify how much a movement adheres to or violates the physical limits of human anatomy. Its significant contribution to the variance explained by the joint-encoding model in EBA (~30%), implies that this body selective region is capable of distinguishing between possible and impossible body movements and also “how impossible” these movement are.

This graded “how impossible?” signal offers a mid-level representational bridge between pose representation and abstract categorical judgments. It not only complements earlier reports that EBA responds to violations of body structure [36] but also resonates with broader theories of embodied cognition, for instance mirror neuron theory of action understanding [72], which posit that perception is grounded in sensorimotor experience. Our findings suggest that the visual system may simulate and test every observed movement against an internal model of human biomechanics [73,71]. This embodied–predictive framework aligns with mirror neuron accounts, where shared motor representations support action understanding [74,75], and with predictive coding framework, where the brain continuously updates predictions about incoming sensory input based on prior knowledge and expectations [76,77]. Specifically, when observing human bodies, the brain may use biomechanical constraints as a basis for these predictions, allowing it to rapidly assess whether a given movement aligns with human motion primitives. By filtering subtle deviations in joint‐angle trajectories, the brain may enhance social perception by rapidly flagging implausible movements that violate physical feasibility [78,77,62,79,80].

### Limitations and future directions

Our scanning parameters focused primarily on occipitotemporal and frontal regions, excluding areas such as motor and premotor cortices. These regions are known to play a crucial role in the recognition of both static and dynamic bodily actions [80,81], responding to biomechanically possible and impossible stimuli [36] and contributing to the distinction between actions that can be performed and those that cannot [78]. Incorporating these regions in future studies will help clarify their role in the perception and discrimination of biomechanical plausibility, offering a more comprehensive view of the neural mechanisms underlying action recognition. Additionally, our stimulus creation was limited to manipulations of the elbows and knees to generate impossible movements. Future research might include a broader range of movements and joint manipulations to evaluate the generality of encoding mechanisms across different biomechanical contexts. Also, the relatively small sample size (n = 11) is common in laminar fMRI studies, but may limit the generalizability of our findings. Replication with larger samples is needed to confirm the observed effects and strengthen the reliability of these results. Finally, further exploration of hemispheric differences, along with the potential influence of attention and task demands on encoding, would enrich our understanding of the factors shaping these neural processes.

## Conclusions

In summary, this study investigated whether occipitotemporal cortex, particularly the body sensitive area EBA, encodes biomechanically possible and impossible body movements. By comparing four encoding models—3D keypoints, similarity distances, categorical differences and motion energy—we found that a combination of these models significantly predicted neural responses in the ventral visual cortex. Notably, the study underscores the brain’s sensitivity to biomechanical plausibility, with the biomechanical (SimDist) model explaining a significant portion of the variance from early stages of visual processing.

Within EBA, the representation holds consistently across inner, middle, and superficial layers in both hemispheres, hinting towards a depth-invariant microcircuit that integrates spatiotemporal, postural, biomechanical, and semantic information.

## Supporting information

S1 Text **Table A.** Pairwise contrasts between encoding models in EBA. We report for each hemisphere and for each pairwise contrast between encoding models, the paired-sample t-statistic (df = 10), the uncorrected p-value, Cohen’s dz effect size (computed as t/√N with N = 11), and the retrospective power at α = 0.05 (two-sided). Positive dz values indicate that the first model in the contrast explained more variance than the second, whereas negative dz values indicate the opposite. **Table B.** Paired-sample t-tests comparing R² values across cortical depths in EBA for each hemisphere (df = 10). For each contrast, we report the uncorrected p-values, q-values (FDR), Cohen’s dz effect size, and the retrospective power at α = 0.05 (two-sided). Negative dz values indicate that the first depth (e.g., inner) had lower R² than the second (e.g., middle or superficial). Power estimates ≥ 0.80 denote adequate sensitivity to detect the observed effects, whereas lower values suggest that non-significant or modest effects may require larger samples or more sensitive methods for reliable detection. **Fig A.** Single-subject prediction accuracy maps. Each panel shows a subject’s cortical surface map of Pearson’s *r* values, obtained by correlating the joint-encoding model’s predicted BOLD time courses (combining motion-energy, 3D keypoints, SimDist, and categorical predictors) with held-out fMRI responses. Model training and testing were performed using 3-fold cross-validation: for each fold, the model was trained on 8 runs (80 stimuli × 6 repetitions) and tested on the remaining 4 runs (40 stimuli × 6 repetitions). Within each training set, data were further split using 4-fold cross-validation (train on 6 runs [60 stimuli × 6 repetitions], validate on 2 runs [20 stimuli × 6 repetitions]). **Fig B.** Single‐subject prediction accuracy map. Same conventions as Fig A. **Fig C.** HSV map of residual variance partitioning for single‐subject results. Hue encodes the relative proportions of variance explained by the three higher-level feature models—3D keypoints (kp3d, red), categorical differences (cat, green), and biomechanical similarity (SimDist, blue)—after factoring out the variance captured by low-level motion energy. Saturation reflects the magnitude of this residual variance (S = 1 − motion-energy fraction), with more saturated colors indicating vertices where higher-level models contribute more strongly. Brightness corresponds to prediction reliability at each vertex (vertex-wise Pearson’s rrr) on the same scale used in the joint-model accuracy maps. **Fig D.** HSV composite map of residual variance partitioning. Same conventions as Fig C.
(PDF)
