# Supplementary material for: Neural encoding of biomechanically (im)possible human movements in occipitotemporal cortex
Source: PLoS Comput Biol. 2025 Dec 8;21(12):e1013694. doi: 10.1371/journal.pcbi.1013694 (PMC12707666; doi:10.1371/journal.pcbi.1013694)
Supplement: S1 Text — Table A. Pairwise contrasts between encoding models in EBA. We report for each hemisphere and for each pairwise contrast between encoding models, the paired-sample t-statistic (df = 10), the uncorrected p-value, Cohen’s dz effect size (computed as t/√N with N = 11), and the retrospective power at α = 0.05 (two-sided). Positive dz values indicate that the first model in the contrast explained more variance than the second, whereas negative dz values indicate the opposite. Table B. Paired-sample t-tests comparing R² values across cortical depths in EBA for each hemisphere (df = 10). For each contrast, we report the uncorrected p-values, q-values (FDR), Cohen’s dz effect size, and the retrospective power at α = 0.05 (two-sided). Negative dz values indicate that the first depth (e.g., inner) had lower R² than the second (e.g., middle or superficial). Power estimates ≥ 0.80 denote adequate sensitivity to detect the observed effects, whereas lower values suggest that non-significant or modest effects may require larger samples or more sensitive methods for reliable detection. Fig A. Single-subject prediction accuracy maps. Each panel shows a subject’s cortical surface map of Pearson’s r values, obtained by correlating the joint-encoding model’s predicted BOLD time courses (combining motion-energy, 3D keypoints, SimDist, and categorical predictors) with held-out fMRI responses. Model training and testing were performed using 3-fold cross-validation: for each fold, the model was trained on 8 runs (80 stimuli × 6 repetitions) and tested on the remaining 4 runs (40 stimuli × 6 repetitions). Within each training set, data were further split using 4-fold cross-validation (train on 6 runs [60 stimuli × 6 repetitions], validate on 2 runs [20 stimuli × 6 repetitions]). Fig B. Single‐subject prediction accuracy map. Same conventions as Fig A. Fig C. HSV map of residual variance partitioning for single‐subject results. Hue encodes the relative proportions of variance explained by th [file pcbi.1013694.s001.pdf]

Supplementary information for Marrazzo et. al

1 Material and Methods

1.1 Biomechanical model (simdist)

To analyze the similarity between morphed movements and normal movements, we utilized motion capture data corresponding to the stimulus videos used in our experiment. The dataset comprised both modified movements (morphed movements) and original, unmodified movements. To measure the similarity between test movements (both modified and original) and the manifold of normal (original) movements, a Gaussian kernel-based approach was employed. This method quantifies the proximity of motion data in the high-dimensional joint angle space, allowing for a robust assessment of movement similarity. The axis-angle data from all time frames and all original (normal) movements were concatenated to form a comprehensive dataset representing the normal actions manifold. This manifold encompasses the typical joint configurations observed during natural movements across all subjects and actions in the experiment.

1.2 Estimation of the Gaussian Width Parameter

To measure similarity in the high-dimensional joint angle space, we employed a Gaussian kernel that required estimation of the Gaussian width parameter σ. This parameter was crucial for appropriately scaling distances within the kernel function.

1.3 Local variance estimation

For each point x_n in the normal actions manifold, we identified its $N = 8$ nearest neighbors based on Euclidean distance in the joint angle space. Due to the high dimensionality of the data (each point represents the axis-angle components of multiple joints), we utilized a k-d tree algorithm (Ranger) (see `https://www3.`

cs.stonybrook.edu/~algorith/implement/ranger/implement.shtml) for efficient nearest neighbor searches. We calculated the average squared distance to the nearest neighbors for each point to estimate the local density:

$$\text{dist}(n) = \frac{1}{N}\sum_{i=1}^{N} \|x_n - x_{\text{n,i}}\|^2 \tag{1}$$

where $x_{\text{n,i}}$ represents the i-th nearest neighbor of point x_n.

The Gaussian width parameter σ^2 was calculated by taking the average of these local distances across all points in the manifold:

$$\sigma^2 = \text{mean}(\text{dist}(n)) \tag{2}$$

This estimation ensures that σ reflects the typical local variance within the data, allowing the Gaussian kernel to appropriately weigh the contributions of different points based on their proximity.

1.4 Similarity Measure Calculation

To quantify the similarity between each test movement (both modified and original) and the normal actions manifold, we utilized a Gaussian kernel-based similarity measure.

For a test movement with T frames and joint data $\{x_{\text{test}}(t)\}_{t=1}^{T}$, the similarity measure S was computed as:

$$S = \sum_{n=1}^{N_{\text{orig}}}\sum_{t=1}^{T} \exp\left(-\frac{\|x_{\text{test}}(t) - x_n\|^2}{2\sigma^2}\right) \tag{3}$$

where:

- N_{orig} is the number of points in the normal actions manifold, namely all time points in all normal actions trajectories.
- x_n are the points in the normal actions manifold.
- $\|\cdot\|$ denotes the Euclidean norm in the joint angle space and σ as in (2).

To normalize the similarity measure and transform it into a distance metric, we defined the similarity distance as:

$$\text{simdist} = -\log\left(\frac{S}{N_{\text{orig}} \times T}\right) \tag{4}$$

This formulation yields a positive value where lower similarity distances indicate a higher similarity between the test movement and the normal actions manifold.

2 Results

2.1 Behavioral analysis

The analysis of the questionnaire responses showed that for the "possible" stimuli, 98% (59 out of 60) were recognized as daily actions, with median ratings between 6 and 7. Conversely, 93% of the "impossible" stimuli (56 out of 60) were not associated with any action, receiving median ratings between 1 and 3. The remaining four stimuli had a median rating of 4. Participants reported focusing on limb movements and parts (arms, legs, hands or feet) in 96.67% of the "impossible" videos (58 out of 60), while 3.33% (2 out of 60) of the videos focused on overall body movement. In contrast, the "possible" videos showed an increased focus on overall body movement (16.67%, 10 out of 60), though the majority still concentrated on limb movements and parts (83.33%, 50 out of 60).

2.2 Statistical Analysis

Table A. The table reports, for each hemisphere and each pairwise contrast between encoding models, the paired-sample t-statistic (degrees of freedom $df = 10$), the uncorrected p-value, Cohen's d_z effect size (computed as $d_z = t/\sqrt{N}$ with $N = 11$), and the retrospective power at $\alpha = 0.05$ (two-sided). Positive d_z values indicate that the first model in the contrast explained more variance than the second, whereas negative values indicate the opposite.

Hemisphere	Contrast	t (df=10)	p_{unc}	q_{FDR}	Effect size (d_z)	Power
RH	Keypoints > Categorical	2.02	0.071	0.086	0.61	0.45
RH	Keypoints > SimDist	–2.65	0.024	0.036	-0.80	0.67
RH	Keypoints > MotEn	–4.99	0.001	0.001	-1.50	0.99
RH	Categorical > SimDist	–5.15	<0.001	0.001	-1.55	1.00
RH	Categorical > MotEn	–7.11	<0.001	<0.001	-2.14	1.00
RH	SimDist > MotEn	–0.75	0.470	0.470	-0.23	0.10
LH	Keypoints > Categorical	1.95	0.080	0.096	0.59	0.42
LH	Keypoints > SimDist	–1.27	0.232	0.232	-0.38	0.21

Continued on next page

Table A – continued from previous page

Hemisphere	Contrast	t (df=10)	p_{unc}	q_{FDR}	Effect size (d_z)	Power
LH	Keypoints > MotEn	–2.91	0.016	0.031	-0.88	0.75
LH	Categorical > SimDist	–5.00	0.001	0.002	-1.51	0.99
LH	Categorical > MotEn	–9.00	<0.001	<0.001	-2.71	1.00
LH	SimDist > MotEn	–2.44	0.035	0.052 -	0.74	0.60

Table B. Paired-sample t-tests comparing R^2 values across cortical depths in EBA for each hemisphere (df=10). We report uncorrected p-values, FDR-corrected q-values, Cohen's d_z (computed as $t/\sqrt{N}$ with $N = 11$), and retrospective power at $\alpha = 0.05$ (two-sided). Negative d_z values indicate that the first depth in the contrast explained less variance than the second.

Hemisphere	Contrast	t (df=10)	p_{unc}	q_{FDR}	Effect size (d_z)	Power
RH	Inner > Middle	−4.01	0.002	0.007	−1.21	0.95
	Inner > Superficial	−2.46	0.034	0.051	−0.74	0.60
	Middle > Superficial	−1.08	0.306	0.306	−0.33	0.17
LH	Inner > Middle	−1.36	0.205	0.307	−0.41	0.23
	Inner > Superficial	−1.36	0.203	0.307	−0.41	0.23
	Middle > Superficial	0.05	0.960	0.960		

3 Single-Subject Results

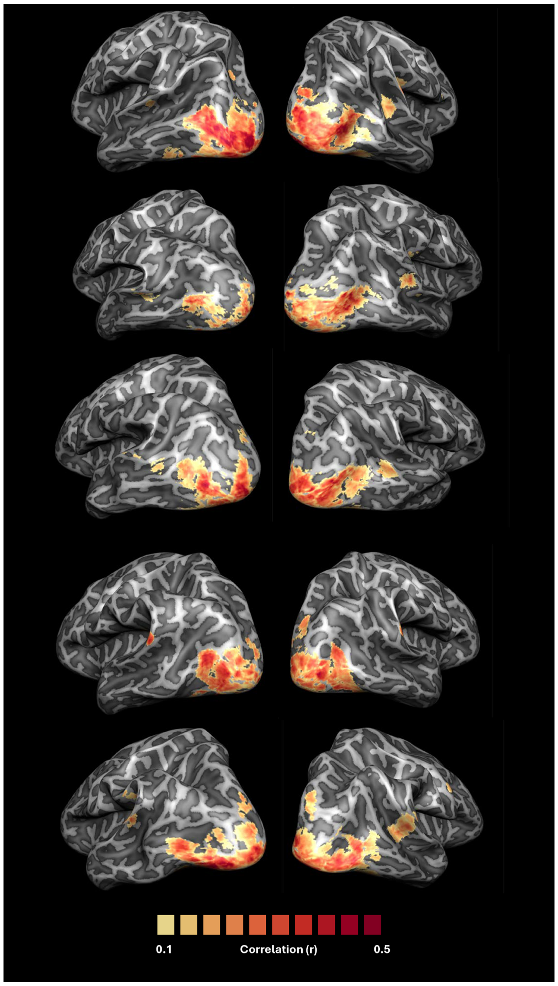

Figure A: **Single-Subject Prediction Accuracy Maps.** Each panel shows a subject's cortical surface map of Pearson's r values, obtained by correlating the joint-encoding model's predicted BOLD time courses (combining motion-energy, 3D keypoints, SimDist, and categorical predictors) with held-out fMRI responses. Model training and testing were performed in cross-validation (3-folds: training on 8 runs [80 stimuli repeated 6 times] and testing on 4 runs [40 repeated 6 times]). For each fold, the training data were additionally split in training set and validation set (4-folds: train on 6 runs [60 stimuli repeated 6 times] and test on 2 runs [20 stimuli repeated 6 times]).

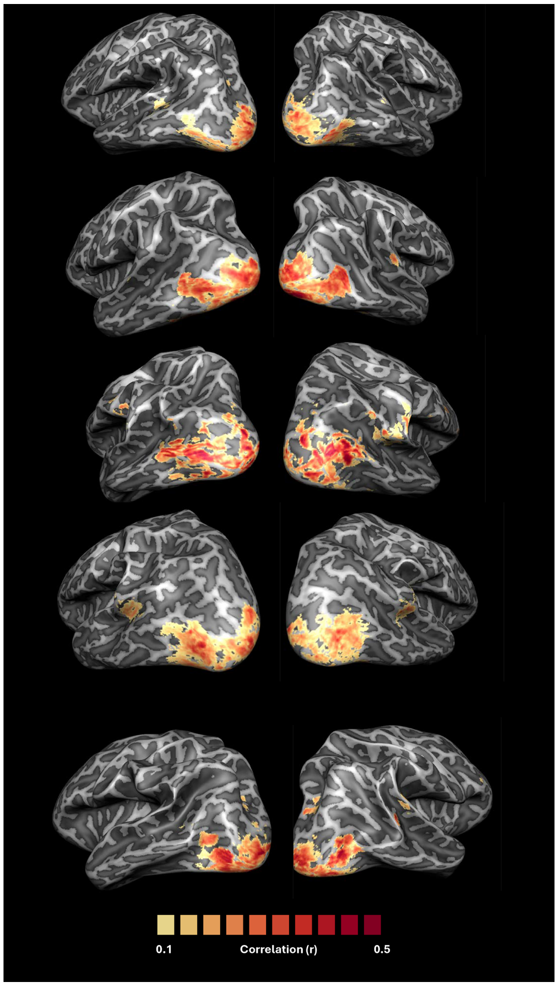

Figure B: **Single-Subject Prediction Accuracy Maps.** Each panel shows a subject's cortical surface map of Pearson's r values, obtained by correlating the joint-encoding model's predicted BOLD time courses (combining motion-energy, 3D keypoints, SimDist, and categorical predictors) with held-out fMRI responses. Model training and testing were performed in cross-validation (3-folds: training on 8 runs [80 stimuli repeated 6 times] and testing on 4 runs [40 repeated 6 times]). For each fold, the training data were additionally split in training set and validation set (4-folds: train on 6 runs [60 stimuli repeated 6 times] and test on 2 runs [20 stimuli repeated 6 times]).

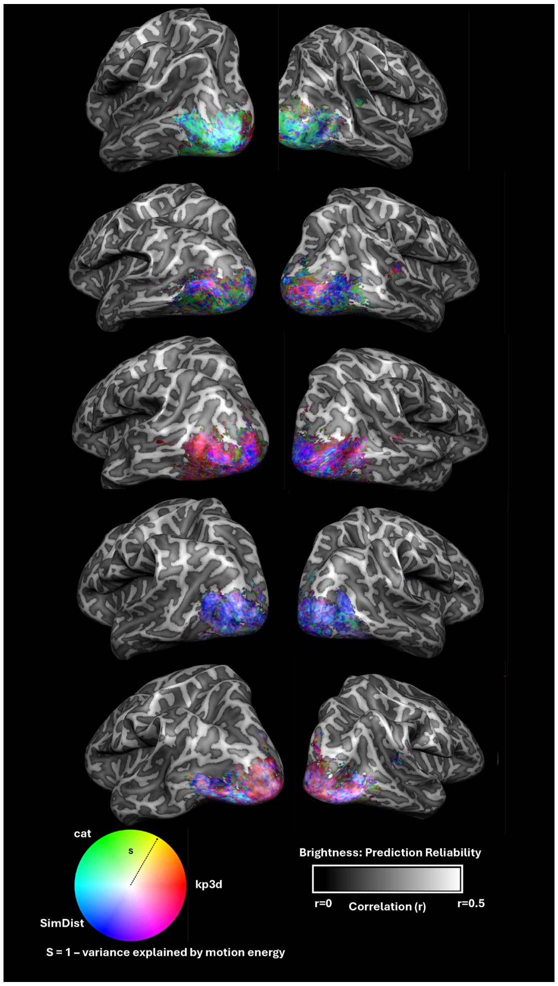

Figure C: **HSV map of residual variance partitioning for Single-Subject results.** Hue encodes the relative proportions of variance explained by the three higher-level feature models—3D keypoints (kp3d, red), categorical differences (cat, green), and biomechanical similarity (SimDist, blue)—after factoring out motion-energy variance. Saturation (colorfulness) reflects the total residual strength, defined as $S = 1-$ (motion-energy fraction). Brightness corresponds to prediction reliability (vertex-wise Pearson's r) on the same scale as the joint-model accuracy maps.

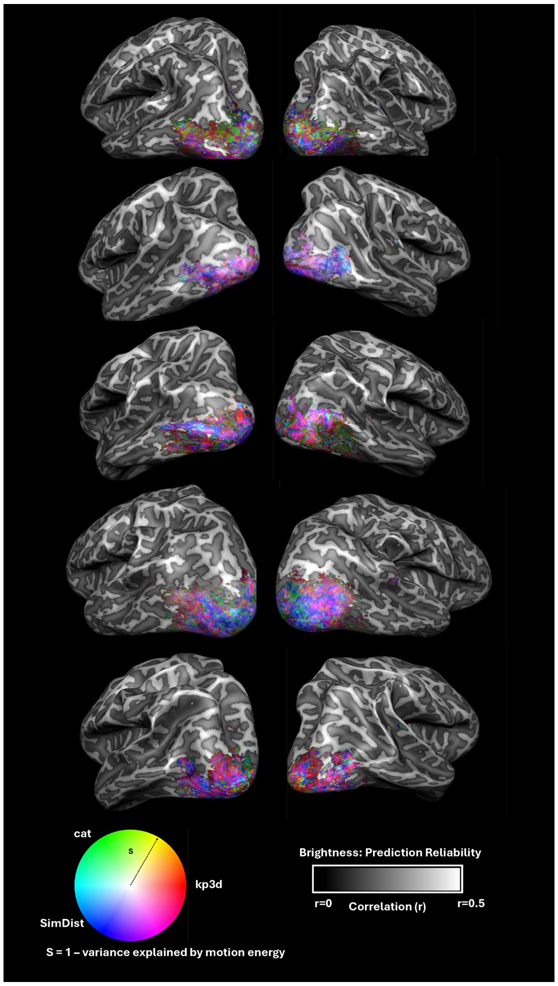

Figure D: **HSV map of residual variance partitioning for Single-Subject results.** Hue encodes the relative proportions of variance explained by the three higher-level feature models—3D keypoints (kp3d, red), categorical differences (cat, green), and biomechanical similarity (SimDist, blue)—after factoring out motion-energy variance. Saturation (colorfulness) reflects the total residual strength, defined as $S = 1-$ (motion-energy fraction). Brightness corresponds to prediction reliability (vertex-wise Pearson's r) on the same scale as the joint-model accuracy maps.
